# Brain-Derived Neurotrophic Factor (BDNF) Regulates Rab5-Positive Early Endosomes in Hippocampal Neurons to Induce Dendritic Branching

**DOI:** 10.3389/fncel.2018.00493

**Published:** 2018-12-17

**Authors:** Guillermo Moya-Alvarado, Andres Gonzalez, Nicolas Stuardo, Francisca C. Bronfman

**Affiliations:** Department of Physiology, Faculty of Biological Sciences, Center for Aging and Regeneration (CARE UC), Pontificia Universidad Católica de Chile, Santiago, Chile

**Keywords:** Neurotrophins, BDNF, TrkB, Rab5, Rab11, endosomes, dendritic branching, neuron

## Abstract

Neurotrophin receptors use endosomal pathways for signaling in neurons. However, how neurotrophins regulate the endosomal system for proper signaling is unknown. Rabs are monomeric GTPases that act as molecular switches to regulate membrane trafficking by binding a wide range of effectors. Among the Rab GTPases, Rab5 is the key GTPase regulating early endosomes and is the first sorting organelle of endocytosed receptors. The objective of our work was to study the regulation of Rab5-positive endosomes by BDNF at different levels, including dynamic, activity and protein levels in hippocampal neurons. Short-term treatment with BDNF increased the colocalization of TrkB in dendrites and cell bodies, increasing the vesiculation of Rab5-positive endosomes. Consistently, BDNF increased the number and mobility of Rab5 endosomes in dendrites. Cell body fluorescence recovery after photobleaching of Rab-EGFP-expressing neurons suggested increased movement of Rab5 endosomes from dendrites to cell bodies. These results correlated with the BDNF-induced activation of Rab5 in dendrites, followed by increased activation of Rab5 in cell bodies. Long-term treatment of hippocampal neurons with BDNF increased the protein levels of Rab5 and Rab11 in an mTOR-dependent manner. While BDNF regulation of Rab5a levels occurred at both the transcriptional and translational levels, Rab11a levels were regulated at the translational level at the time points analyzed. Finally, expression of a dominant-negative mutant of Rab5 reduced the basal arborization of nontreated neurons, and although BDNF was partially able to rescue the effect of Rab5DN at the level of primary dendrites, BDNF-induced dendritic branching was largely reduced. Our findings indicate that BDNF regulates the Rab5-Rab11 endosomal system at different levels and that these processes are likely required for BDNF-induced dendritic branching.

## Introduction

Brain-derived neurotrophic factor (BDNF) is a well-known neurotrophin that belongs to a small family of secreed proteins that includes nerve growth factor (NGF), neurotrophin-3 (NT3) and neurotrophin-4 (NT4) (Park and Poo, 2013). BDNF regulates many facets of the central neurons, including neuronal survival and differentiation, neuronal growth, synaptogenesis and plasticity and maintenance of neuronal circuits. BDNF is the most widely expressed neurotrophic factor in the brain and exerts its function by binding to the tropomyosin-related kinase receptor TrkB and the p75 neurotrophin receptor (p75). In addition, BDNF is secreted in an activity-dependent manner by autocrine and paracrine mechanisms (Bronfman et al., 2014).

The neuronal growth effects mediated by BDNF are mainly mediated by its tyrosine kinase receptor TrkB (Gonzalez et al., 2016). For example, BDNF binding to TrkB increases the branching of cortical and hippocampal neurons in dissociated cultures and organotypic slices (Horch and Katz, 2002). In addition, BDNF regulates the survival and migration of cortical neurons (Zhou et al., 2007; Zheng et al., 2008). These effects are induced by the activation of downstream signaling pathways after BDNF/TrkB receptor interaction. After binding BDNF, TrkB dimerizes and undergoes autophosphorylation at specific tyrosine residues of the intracellular domain. These phosphotyrosines are docking sites for adaptor proteins that lead to the activation of several signaling cascades including the mitogen-activated protein kinases (MAPKs), such as ERK1/2, ERK5 and p38, in addition to the phosphatidylinositol-3-kinase (PI3K)-Akt-mTOR pathway, phospholipase C-γ (PLC-γ) and the small GTPases of the Rho family Cdc42/Rac/RhoA (Huang and Reichardt, 2003; Minichiello, 2009).

Different lines of investigation have shown that internalization and postendocytic trafficking of Trk receptors determine their signaling properties and thus functional outcomes in neurons (Bronfman et al., 2014; Cosker and Segal, 2014). For example, Trk receptors ensure localized signaling responses to extracellular cues in axons (Ascano et al., 2012) and enhance downstream signaling to regulate neuronal differentiation (Zhang et al., 2000) and dendritic arborization (Lazo et al., 2013). Additionally, BDNF signals are retrogradely transported from dendrites to the soma to regulate gene expression (Cohen et al., 2011). Internalization of BDNF/TrkB is required for the sustained activation of PI3K and ERK signaling pathways and neurite outgrowth (Kumar et al., 2005; Zheng et al., 2008). Additionally, after internalization, endocytosed TrkB recruits microtubule-associated molecular motors such as dynein and neuronal kinesin KIF21B, which have both been described to contribute to the directionality of BDNF/TrkB endosomes in dendrites (Ghiretti et al., 2016; Ayloo et al., 2017).

The Rab monomeric GTPases are the main regulators of postendocytic trafficking of endocytic receptors. Rabs act as key regulators of vesicular trafficking by controlling the transport, anchoring and coupling of vesicles through effector binding. Among these effectors are the molecular motors and the SNARES, which generally join the Rabs in their GTP-bound state (Grosshans et al., 2006; Stenmark, 2009). In fact, Rabs are mediators of TrkB endosomal signaling (Zhou et al., 2012; Lazo et al., 2013; Sui et al., 2015). In the literature, more than 60 members of the GTPase Rab family have been described (Stenmark, 2009). Rab5, Rab7 and Rab11 are among the key GTPases known to be involved in BDNF/TrkB signaling (Zhou et al., 2012; Lazo et al., 2013).

After internalization, tyrosine kinase receptors (TRKs) enter the early or sorting endosomes, whose biology is regulated by Rab5 (Goh and Sorkin, 2013). Independent of the internalization mechanism of receptors, Rab5 tightly regulates the homotypic fusion of endosomes, forming the early or sorting endosome (Stenmark, 2009). There, receptors are sorted to the recycling pathway, which is regulated by Rab11, or to the late endocytic pathway regulated by Rab7 (Bronfman et al., 2014).

Studies from our laboratory and others have established that BDNF/TrkB regulates the activity and dynamics of Rab11-positive endosomes; in turn, Rab11 is required for BDNF-induced dendritic branching and local signaling in dendrites and synapses (Huang et al., 2013; Lazo et al., 2013; Song et al., 2015; Sui et al., 2015). Thus, transit through the early recycling pathway of TrkB receptors is a key step in BDNF signaling in neurons. However, whether BDNF/TrkB regulates Rab5 activity and dynamics in dendrites is unknown. Several lines of evidence indicate that Rab5-positive endosomes are required for proper neuronal morphology. Genetic experiments in Drosophila have shown that dynein and Rab5 are required for dendritic arborization in larvae (Satoh et al., 2008). On the other hand, Rab5 activity is regulated by TrkA in PC12 differentiation assays (Liu et al., 2007). Here, we first studied the short-term effects of BDNF treatment (5–30 min) on Rab5 dynamics and activity and then the long-term effects of BDNF treatment (4–24 h) on Rab5 and Rab11 protein and mRNA levels. We found that BDNF increases the number and dynamics of Rab5-positive endosomes in dendrites. Indeed, fluorescence recovery after photobleaching (FRAP) experiments showed that BDNF increases the recovery of Rab5-positive vesicles in the soma, which correlates with the increased activity of somatic Rab5, suggesting that BDNF increases the activation and movement of dendritic endosomes to cell bodies. Long-term treatment of hippocampal neurons with BDNF increased the protein levels of both Rab5 and Rab11 in an mTOR-dependent manner. In addition, BDNF also regulated mRNA levels of *rab5* (but not the mRNA levels of *rab11*). Both, Rab5 and Rab11 activity was required for proper morphological changes induced by long-term BDNF (48 h) treatment of neurons. Of note, in contrast to Rab11, reduced Rab5 activity impacted the basal levels of primary dendrites. BDNF was partially able to rescue this effect, but reduced Rab5 activity halted the full dendritic arborization induced by BDNF. Altogether, these results suggest that BDNF regulates the early recycling pathway at different levels to induce dendritic branching.

## Methodology

All experiments were carried out in accordance with the approved guidelines of CONICYT (Chilean National Commission for Scientific and Technological Research). The protocols used in this study were approved by the Biosecurity and Bioethical and Animal Welfare Committees of the P. Catholic University of Chile. Experiments involving vertebrates were approved by the Bioethical and Animal Welfare Committee of the P. Catholic University of Chile.

### Materials

Minimum essential medium (MEM, 11700-077), Dulbecco's Modified Eagle's Medium (DMEM, 12800-017), Hank's balanced salt solution (HBSS, 14065-056), neurobasal medium (21103-049), OptiMEM (11058-021), Lipofectamine 2000 (11668-027), glutamine, B27 (17504-044), horse serum (HS, 16050-122), penicillin/streptomycin (15140-148), and trypsin (15090-046) were obtained from Invitrogen (Life Technologies, CA, US). Fetal bovine serum (FBS) HyClone (SH30071.03) was from GE Healthcare Life Science. Poly-L-lysine (P2636), AraC, Glutathione-Sepharose 4B (GE17-0756-01) and isopropyl β-D-thiogalactoside (IPTG, I6758) were from Sigma (MO, US). BDNF was purchased from Alomone Labs (Jerusalem, Israel). TrkB-Fc was acquired from R&D Systems (688TK, MN, US). Anti-βIII tubulin antibody, mouse anti-Flag (F3165), Mowiol 4-88 and the inhibitor actinomycin D (A1410) were purchased from Sigma (St. Louis, MO, US). Protease-free bovine serum albumin (BSA) was purchased from Jackson ImmunoResearch (West Grove, PA, US). A MAP2 antibody was purchased from Upstate-Millipore (Billerica, MA). Protein-phosphatase inhibitors were from Thermo Fisher Scientific. The inhibitors cycloheximide (239763) and rapamycin (553210) were purchased from Calbiochem (Darmstadt, Germany). Mouse anti-glutathione-S-transferase (GST) (Ab92) and mouse anti-Rab5 (ab18211) were purchased from Abcam. Rabbit anti-Rab11 (715300) was purchased from Invitrogen. The Flag-TrkB plasmid was a gift of Dr. Francis Lee (Weill Cornell University, NY, US), EGFP-Rab5 and EGFP-Rab5DN were gifts of Dr. Victor Faundez (Emory University, GA, US), EGFP- Rab11DN was a gift of Dr. Rejji Kuruvilla (John Hopkins University, MD, US), and pGEX-GST-Rabaptin5 was donated by Dr. Vicente Torres (University of Chile, Chile).

### Hippocampal Neuron Primary Culture

Embryonic hippocampal neurons from rats of either sex (embryonic days 17-19) were dissected as described previously (Shimada et al., 1998; Fan et al., 2004) in HBSS. After disaggregation, neurons were resuspended in MEM supplemented with 10% HS, 20% D-glucose, and 0.5 mM glutamine and were seeded on coverslips or plastic plates coated with poly-L-lysine (1 mg/ml). For morphological experiments, 7000 cells/cm^2^ were seeded on coverslips. For protein or mRNA experiments, 15000 cells/cm^2^ were seeded on plastic plates. After 4 h, the culture medium was replaced with neurobasal medium supplemented with 2% B27 and 1X glutamax. Proliferation of nonneuronal cells was limited using cytosine arabinoside at 3 days *in vitro* (DIV). The animals were obtained from the animal facilities of Pontificia Universidad Católica de Chile and euthanatized under deep anesthesia according to the bioethical protocols of our institution.

### Analysis of the Levels of Messenger RNA (mRNA) in Hippocampal Neurons After BDNF Stimulation

Hippocampal neurons at 9 DIV were incubated for 90 min in neurobasal media for depletion of endogenous trophic factors and then were treated with 50 ng/mL BDNF for 4 or 12 h. Total RNA was extracted from primary neurons by using TRIzol and purified using the RNeasy kit (Qiagen, Hilden, Alemania) according to the manufacturer's instructions. cDNA was prepared by reverse transcription of 1 μg of total RNA with random primers using Maloney Murine Leukemia Virus Reverse Transcriptase (M-MLV RT, Promega). The resulting cDNAs were amplified by using Brilliant II SYBR Green qPCR (Stratagene) with an Mx3000P thermocycler (Stratagene). All mRNA expression data were normalized to β*-actin, tbp* and *pjk-1* expression in the corresponding sample (Santos and Duarte, 2008). Finally, 2^−ΔΔ*Ct*^ analysis was performed. Oligonucleotide sequences for the primers used are shown in Table 1.

**Table 1:** Primers used to evaluate the mRNA levels of Rab5a, Arc, β-actin, TBP, and PGK-1.

**Gene**	**Primer (5^**′**^-3^**′**^)**
*rab5a*	F:GGCTAATCGAGGAGCAACAA
	R:ACAAAGCGAAGCACCAGACT
*arc*	F:GGAGGGAGGTCTTCTACCGT
	R:CTACAGAGACAGTGTGGCGG
*β-actin*	F:CCCGCGAGTACAACCTTCT
	R:CGTCATCCATGGCGAACT
*tbp*	F:CTGTTTCATGGTGCGTGACGAT
	R:AAGCCCTGAGCATAAGGTGGAA
*pgk-1*	F:TGCTGGGCAAGGATGTTCTGTT
	R:ACATGAAAGCGGAGGTTCTCCA
*rab11a*	F:AAAGTTACCCTGCTGCCTGG
	R:CTGCCAGGAAAGGAGACTGG

*F, forward primer; R, reverse primer*.

### Western Blot Analyses

To study Rab5a and Rab11a protein levels, neurons were depleted with neurobasal media in the presence or absence of 5 μM actinomycin D (for Rab5a) or 25 μM cycloheximide (for Rab5a and Rab11a) with 50 ng/mL BDNF for 24 h or were treated in the presence or absence of 200 nM rapamycin for 60 min and then stimulated for 4 and 12 h with 50 ng/mL BDNF in the presence or absence of the drug. Next, cells were lysed with lysis buffer (150 mM NaCl, 50 mM Tris-HCl pH 8.0, 2 mM EDTA, 0.1% SDS and 1% Triton X-100) containing protease and phosphatase inhibitors. Standard SDS gel electrophoresis and Western blotting procedures were used to analyze the cell extracts using anti-Rab5a (1:1000), anti-Rab11a (1:1000) and anti-β-III tubulin (1:1000) antibodies.

### Immunoendocytosis of Flag-TrkB and Colocalization

Neurons were transfected with Flag-TrkB and EGFP-Rab5 using Lipofectamine 2000 and the manufacturer's instructions when cultures were at 7 DIV. Forty-eight hours later, neurons were incubated for 90 min in neurobasal media for depletion of endogenous trophic factors and treated with mouse anti-Flag antibodies conjugated to an Alexa Fluor 555 fluorochrome (20 μg/mL). After 30 min, the cells were washed with PBS at 37°C and stimulated with 50 ng/mL BDNF for 5 or 15 min, fixed and compared with noninternalized controls (cells not treated with BDNF). Images of neurons were acquired using confocal microscopy, processed with deconvolution algorithms, and then colocalization of Flag-TrkB with EGFP-Rab5 was analyzed by calculating Manders correlation index (M1) (Bolte and Cordelières, 2006).

### Live-Cell Imaging of EGFP-Rab5

Neurons were transfected with EGFP-Rab5 as described above. After 24 h, the cells were depleted with neurobasal media during 180 min. Then, the cells were transferred to a Tyrode media (124 mM NaCl, 5 mM KCl, 2 mM CaCl_2_, 1 mM MgCl_2_, 30 mM D-glucose and 25 mM HEPES, pH 7.4). Live-cell imaging was performed on a Nikon Eclipse C2 confocal microscope equipped with a live-cell temperature controller (LCI cu-501) and digital camera connected to a computer with Software NIS-Elements C. Images of a single neuron transfected with EGFP-Rab5 were acquired using a 60X objective at intervals of 7.3 s for 5 min to establish the basal level of distribution and dynamic. After 5 min, neurons were stimulated with 50 ng/mL BDNF, allowing 3 min for diffusion of the ligand, and we started an additional 30 min of capture.

Quantification of the number of endosomes-like vesicles containing EGFP-Rab5 in dendrites was performed by comparing the fraction of total dendritic Rab5 that was found in structures larger than 0.2 μm^2^. Images of the video were segmented with ImageJ, and the number of endosome-like vesicles was quantitated in 30-μm segments of primary dendrites.

Analysis of the mobility of Rab5-positive endosomes was performed by comparing the distribution of fluorescence in the same dendrite at different time points (0, 5, 15, 30 min). We quantified the number of particles moving more than 5 μm as a mobile fraction in nonstimulated neurons (control) and in neurons treated with BDNF for 5-30 min.

### Live-Cell Imaging and Fluorescence Recovery After Photobleaching (FRAP) of EGFP-Rab5

The neurons were transfected with EGFP-Rab5 at 8 DIV. After 24 h, the cells were depleted from B27 for 180 min in neurobasal media. Then, the cells were transferred to a Tyrode media supplemented with TrkB/Fc (200 nM), and live-cell imaging was performed on a Nikon Eclipse C2 confocal microscope equipped with a live-cell temperature controller (LCI cu-501). Images of a single neuron transfected with EGFP-Rab5 were acquired using a 60X objective at intervals of 4 s for 5 min each at 5, 15 and 30 min to establish the basal level. After a brief wash with Tyrode media, neurons were stimulated with 50 ng/mL BDNF, allowing 3 min for diffusion of the ligand, and we started an additional 30 min of capture for intervals of 4 s for 300 s each at 5, 15, and 30 min. For the FRAP assay, a prebleach image was acquired at 2% laser power, after which a selected area was bleached at 100% laser power with 10 successive bleach scans separated by 1 s, assisted by the microscope software. Postbleach recovery images were acquired every 7.3 s for 300 s. Postacquisition image processing was performed using ImageJ. Adjustment and analyses were performed on the videos as brightness/contrast adjustments to all pixels in the images and as manual tracking of objects across multiples frames, respectively (Snapp et al., 2003). To quantify the percentage of endosome-like vesicles in the cell bodies of neurons transfected with EGFP-Rab5, first a threshold of the photobleached zone was applied. Prior to bleaching, a quantification was performed using the same selected region of interest (ROI). Then, the number of vesicles that recovered fluorescence associated with EGFP-Rab5 was quantified at 0, 5, 15, and 30 min after photobleaching.

### Microscopy Detection and Quantification of Active Rab5 in Dendrites and Cell Bodies

The fusion protein Rab5BD-GST was produced in BL21 E. coli, transformed with a pGEX-GST-Rabaptin5 plasmid and stimulated for 4 h with IPTG. The Rab5BD-GST protein was purified from bacteria lysate using glutathione-Sepharose beads. For use as a probe, the protein was eluted in a solution of reduced glutathione. Similar methods have been described previously for other GTPases such as GST-FIP3 (Lazo et al., 2013). To test the protein as a probe, hippocampal neurons at 8 DIV were transfected with EGFP-Rab5DN, EGFP-Rab5CA or EGFP; in addition, nontransfected neurons were stimulated with 50 ng/mL BDNF for 5 or 30 min, fixed with paraformaldehyde (PFA), permeabilized and blocked in 3% fish gelatin in incubation buffer (50 mM Tris-Cl, 50 mM NaCl, 5 mM MgCl_2_, 0.5 mM DTT, 1 mM EDTA, 0.25 M sucrose and 0.2% Triton X-100, pH 7.2) for 45 min. The neurons were then incubated overnight with ~10 μg/mL Rab5BD-GST in incubation buffer at 4°C. After 2 brief washes in HBSS, the neurons were fixed again in PFA, washed in PBS and then a standard immunofluorescence assay with rabbit anti-GST (1:500) and mouse anti-MAP2 (1:1000) was performed.

The neurons to be quantified were selected based on the MAP2 labeling to avoid the specific selection of a neuron with high or low levels of Rab5BD-GST. Three primary dendrites and the cell body were identified, and the integrated intensity was measured (intensity of the signal standardized by the area) per cell body and associated dendrites. The background was calculated from images of neurons treated with GST, and this baseline was calculated for and subtracted from each dataset.

### Stimulation and Measurement of Dendritic Arborization Induced by BDNF

Hippocampal neurons (7 DIV) were transduced with EGFP, EGFP-Rab5DN or EGFP-Rab11DN adenoviruses and stimulated with 50 ng/mL BDNF in culture medium. After 48 h, dendritic arborization was analyzed by Sholl analysis (Sholl, 1953) and by counting the number of branching points as described previously (Lazo et al., 2013). For analysis of dendritic branching, the neurons were immunostained with anti-MAP2. Dendrites were visualized by confocal microscopy using a Zeiss Axiovert 2000 inverted microscope equipped with a laser scanning module and Pascal 5 software (Carl Zeiss). Images were acquired using a 63X objective at 1024 X 1024-pixel resolution along the z-axis of whole cells. Z-stacks were integrated, and the images were segmented to obtain binary images. Ten concentric circles with increasing diameters (10 μm each step) were traced around the cell body, and the number of intersections between dendrites and circles was counted and plotted for each diameter. The adenovirus vector work was performed under biosafety level 2 conditions using a Labculture Class II, Type A2 cabinet (ESCO, Singapore). Analysis was performed using the ImageJ program.

### Statistics

For statistical analysis, the GraphPad Prism 7 program was used. Multiple comparisons were performed with ANOVA with Bonferroni's posttest. To determine if two sets of data were significantly different from each other the Student's *t*-test was applied.

## Results

### BDNF Increases the Colocalization of TrkB With Rab5-Positive Endosomes, Increasing its Vesiculation and Mobility in Dendrites

**Figure 1:**
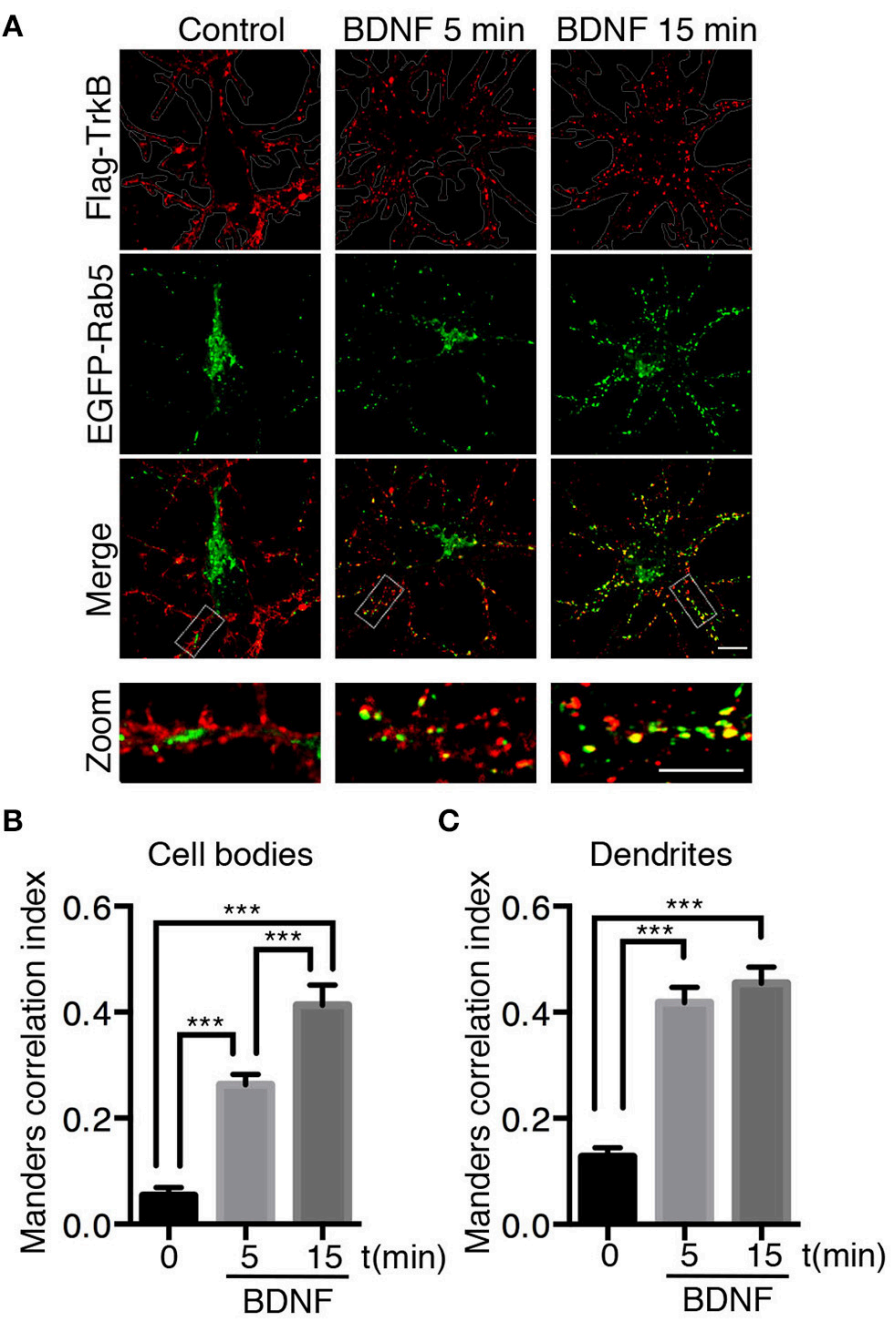
Endocytosed TrkB receptors colocalize with Rab5 after BDNF stimulation in dendrites and cell bodies of hippocampal neurons. Hippocampal neurons (7 DIV) were cotransfected with Flag-TrkB and EGFP-Rab5 (green). After 48 h, neurons were incubated with anti-Flag antibodies (red) at 4°C for 30 min. TrkB internalization was stimulated with BDNF at 37°C for 5 or 15 min and then the neurons were fixed and observed by confocal microscopy. **(A)** In nonstimulated neurons, TrkB receptors are localized in the plasma membrane, and Rab5 is located within the neurons (time 0). After BDNF stimulation, TrkB receptors show an intracellular distribution and colocalize with Rab5. **(B)** Quantification of colocalization of TrkB-Flag with EGFP-Rab5 in cell bodies using Manders correlation index. **(C)** Quantification of colocalization of Flag-TrkB with EGFP-Rab5 in primary dendrites using Manders correlation index. *N* = 30 neurons from 3 different experiments. The results are expressed as the mean ± SEM. ***p < 0.001 by ANOVA with Bonferroni's posttest.

Neurotrophins use the early endosomal route, regulated by the Rab5 monomeric GTPase, to signal and regulate different physiological processes (Deinhardt et al., 2006; Ascano et al., 2012; Lazo et al., 2013). However, to date, there are no studies addressing the functional relationship of Rab5-positive endosomes with BDNF signaling. To address this issue, we analyzed the dynamics and activity of Rab5 endosomes upon short-term administration (5–30 min) of BDNF. First, we studied whether TrkB and Rab5 colocalize after BDNF treatment by cotransfecting hippocampal neurons with EGFP-Rab5 and Flag-TrkB tagged on its NH2 domain. As reported previously for the colocalization of Rab11 and TrkB, we performed immunoendocytosis by labeling the surface expression of TrkB in the absence or presence of BDNF (Lazo et al., 2013). We observed that on neurons that were not stimulated with BDNF, the TrkB receptors were dispersed to the periphery of the cell bodies and dendrites in large patches (Figure 1A). In addition, EGFP-Rab5 was concentrated in the cell body, although it was possible to identify some Rab5-positive endosomes in the dendrites (Figure 1A). After stimulation with BDNF, the EGFP-Rab5 distribution was more vesiculated, and there was an apparent increase in the presence of EGFP-Rab5 in dendrites. TrkB distribution also appeared more vesiculated after 15 min of BDNF treatment (Figure 1A). In addition, BDNF increased the colocalization of TrkB-positive endosomes with EGFP-Rab5 endosomes in cell bodies in a time-dependent manner (Figure 1B), as well as in dendrites (Figure 1C). Interestingly, after 5 min of BDNF treatment, the colocalization of TrkB and Rab5 in dendrites was already the same as at 15 min of stimulation. However, in the soma, the colocalization was increased after 15 min of treatment compared to that at 5 min (Figures 1B,C). These results suggest that BDNF increases TrkB and Rab5 colocalization and changes the dynamics of Rab5 in the dendrites and somas of hippocampal neurons.

**Figure 2:**
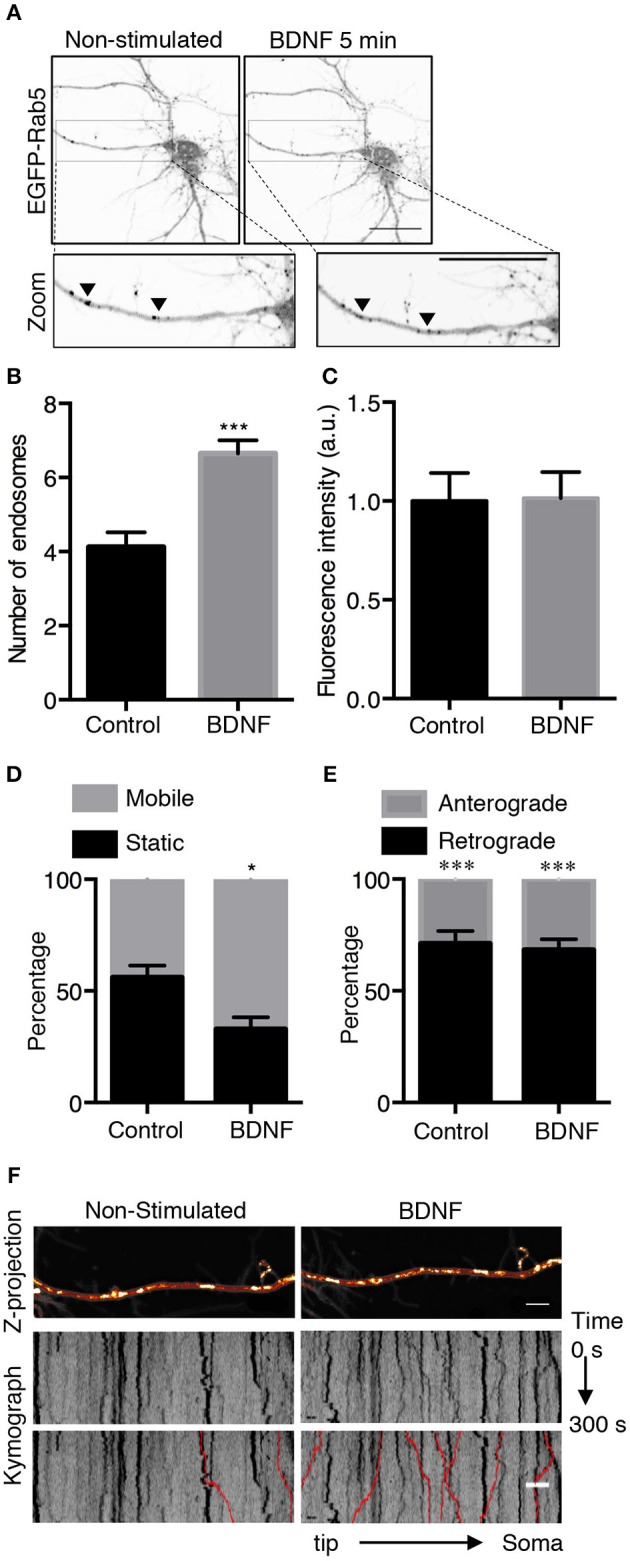
BDNF increases the number and mobility of Rab5-positive endosomes in dendrites. **(A)** Representative image illustrating changes in the number of Rab5-positive endosomes in nonstimulated conditions (control) and after 5 min of BDNF treatment (BDNF) in the same neuron. EGFP-Rab5 endosomes are observed as dark spots within dendrites. A zoomed-in image of the dendrite is shown in the lower part, indicating that BDNF increases the number of Rab5-positive endosomes in dendrites (arrowheads). Scale bar, 10 μm. **(B)** Quantification of the number of endosomes in 30 μm^2^ of dendrites in nonstimulated neurons (control) and after BDNF treatment (5 min). Endosomes were segmented by a fluorescence threshold and then quantified using ImageJ software. A total of 40 dendrites from 6 neurons were included in the study from 3 independent experiments. **(C)** Quantification of the fluorescence intensity of EGFP-Rab5 in the dendrites of neurons in the nonstimulated and BDNF conditions. **(D)** Quantification of mobile and static particles in dendrites expressed as a percentage based on the total number of particles per condition. Endosomes that traveled 5 μm or more after 300 s of recording were considered mobile endosomes. **(E)** Quantification of anterograde and retrograde mobile particles in dendrites expressed as a percentage of the total number of particles in each condition. **(F)** Representative image of a Z-projection of dendrites in the nonstimulated and BDNF conditions, showing the change in the mobile fraction of EGFP-Rab5. In the lower part is the kymograph of each neurite during the 300 s recording. In red are the endosomes considered to be mobile vesicles. Scale bar, 5 μm. The results are expressed as the mean ± SEM. ^*^p < 0.5 or ^***^p < 0.001 by Student's *t*-test.

To further study whether BDNF regulates Rab5-positive endosomes, we studied the dynamics of EGFP-Rab5 in transfected hippocampal neurons by time-lapse microscopy of living cells before and after 5 min of BDNF stimulation. We found an increase in the number of Rab5-positive endosomes (Figures 2A,B), defined as dark vesicles using a threshold analysis in ImageJ, without changing the total EGFP-Rab5-associated fluorescence (Figure 2C). Additionally, the mobility of Rab5 after BDNF treatment was increased, measured as endosomes that moved more than 5 μM in a time lapse of 300 s, shown as red lines in the kymograph (Figures 2D,F). Interestingly, the movement of EGFP-Rab5 is biased to the retrograde direction, as reported before in the literature (Kollins et al., 2009; Ayloo et al., 2017), a process that was not changed with the addition of BDNF (Figure 2E).

### BDNF Increases the Recovery of Vesicular Rab5 After Photobleaching in the Cell Body, a Process That Correlates With Increased Rab5 Activity

**Figure 3:**
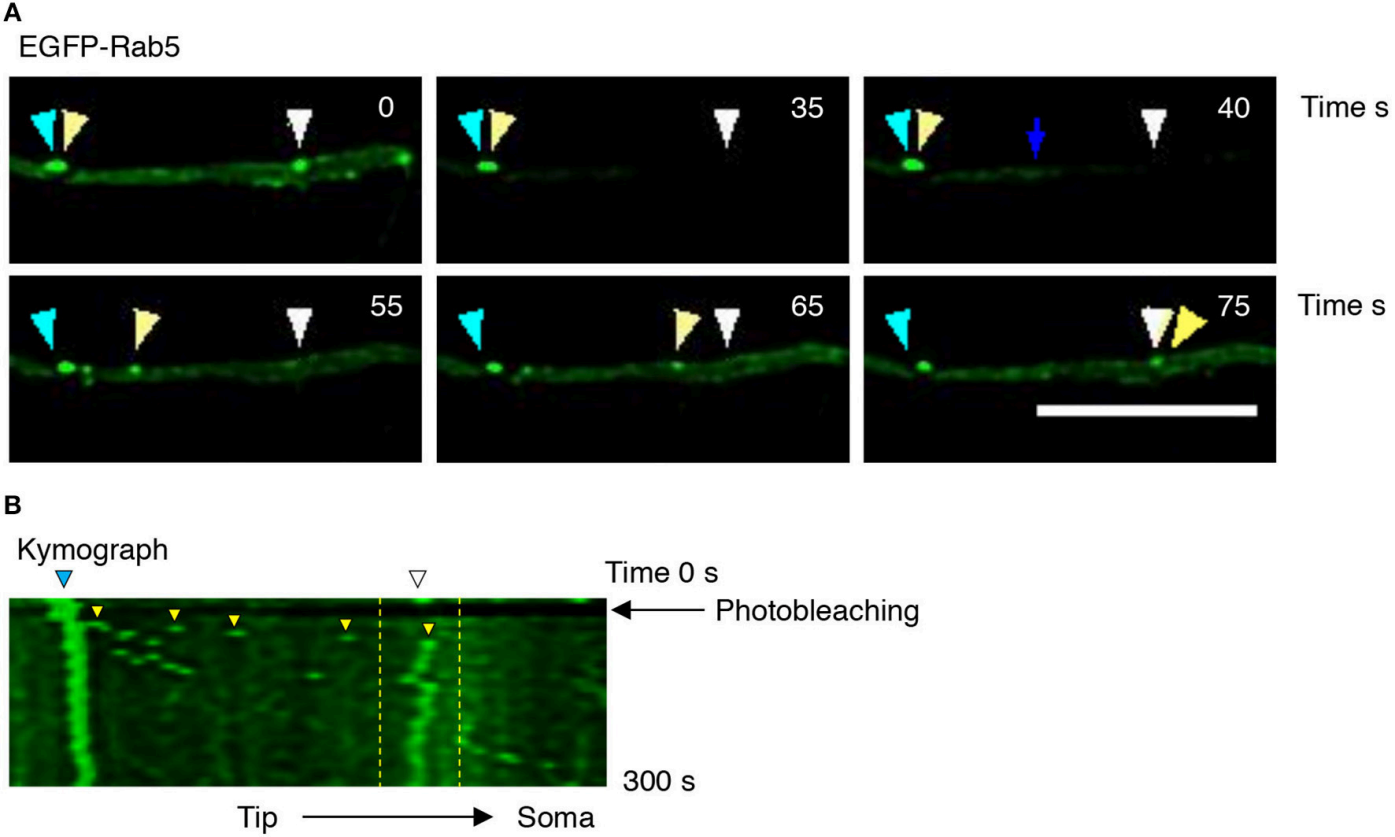
Evaluation of EGFP-Rab5 movement by time-lapse microscopy after photobleaching in hippocampal neurons treated with BDNF. **(A)** Representative image of FRAP performed in primary dendrites of hippocampal neurons treated with BDNF. The recovery was evaluated during 300 s. Images were taken every 7.3 s. Representative individual frame of time-lapse performed in a neuron expressing EGFP-Rab5. The image shows both cytosolic-associated EGFP-Rab5 and vesicular-associated EGFP-Rab5 fluorescence (green) in dendrites prior to FRAP (0 s), during FRAP (35 s), and after photobleaching (until 75 s). The blue arrow shows nonvesicular (or soluble) EGFP-Rab5 fluorescence recovery. The white arrowhead indicates a static endosome that is photobleached (35 s), which is recovered after 40 s by recruiting a mobile endosome, which is indicated with the yellow arrowhead. The cyan arrowhead shows a static endosome that generates the endosome labeled with a yellow arrowhead. Scale bar, 10 μm. **(B)** Kymograph of the endosome movement event shown in **(A)**. In between the yellow lines is located the endosome recovered **(**indicated by the white arrowhead in **A)** by a retrograde-transported endosome, which is indicated by the yellow arrowhead in **(A)**. The black arrow indicates the moment in which the photobleaching was performed (10 s).

**Figure 4:**
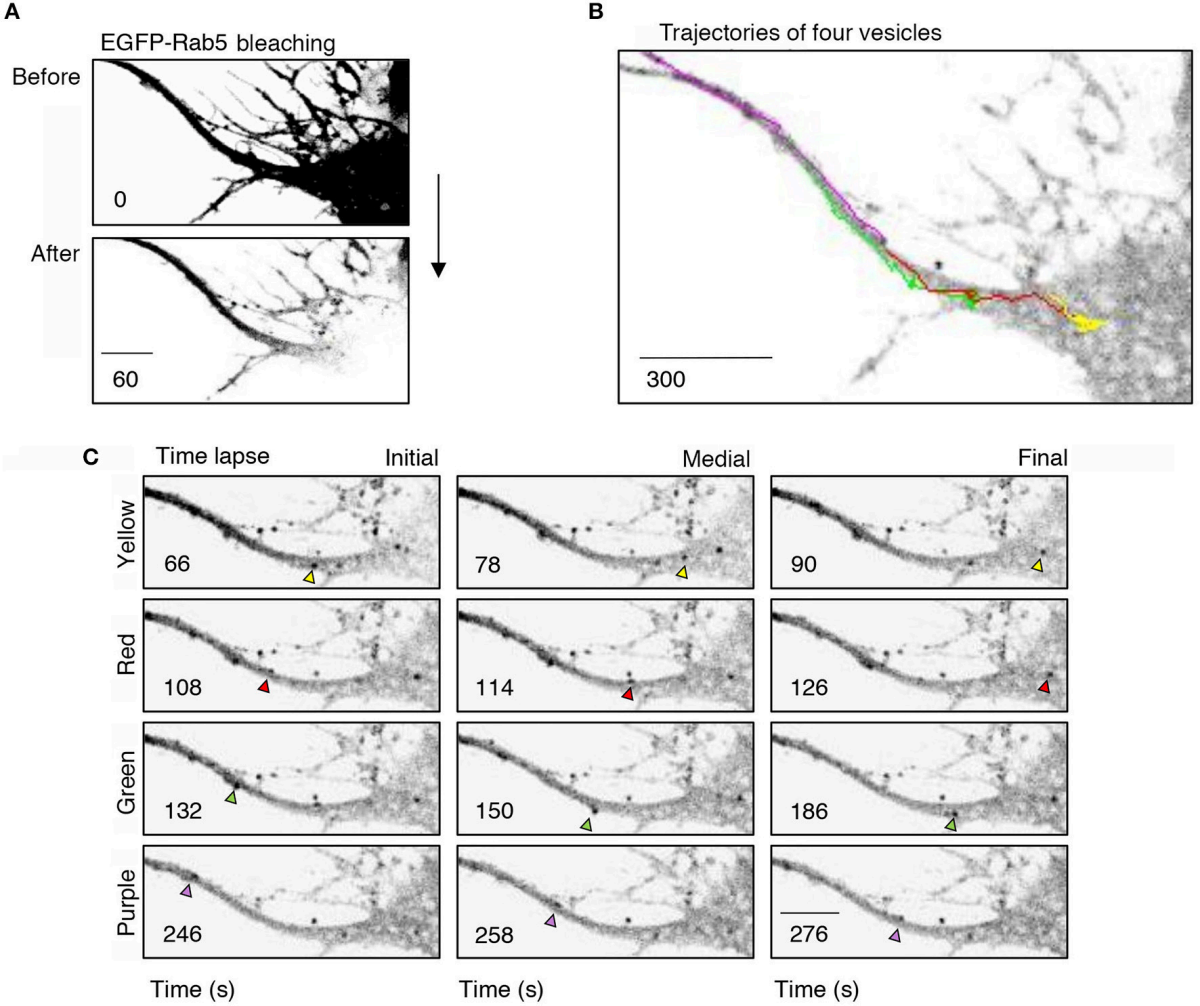
Retrograde transport of EGFP-Rab5 endosomes from dendrites to the cell body. **(A)** Cell body and primary dendrite of a hippocampal neuron before (upper panel) and after photobleaching (lower panel). **(B)** Representative image of a time-lapse recording of EGFP-Rab5-associated fluorescence after photobleaching indicating the trajectories of four vesicles positive for EGFP-Rab5 performed in a primary dendrite and soma of a hippocampal neuron stimulated with BDNF. **(C)** Representative image of the initial, medial and final point of the trajectories shown in **(B)**. The vesicles whose trajectories were labeled in **(B)** are indicated with arrowheads of the same color of the trajectory. The numbers inside the panels indicate the seconds after photobleaching. Scale bar, 10 μm.

**Figure 5:**
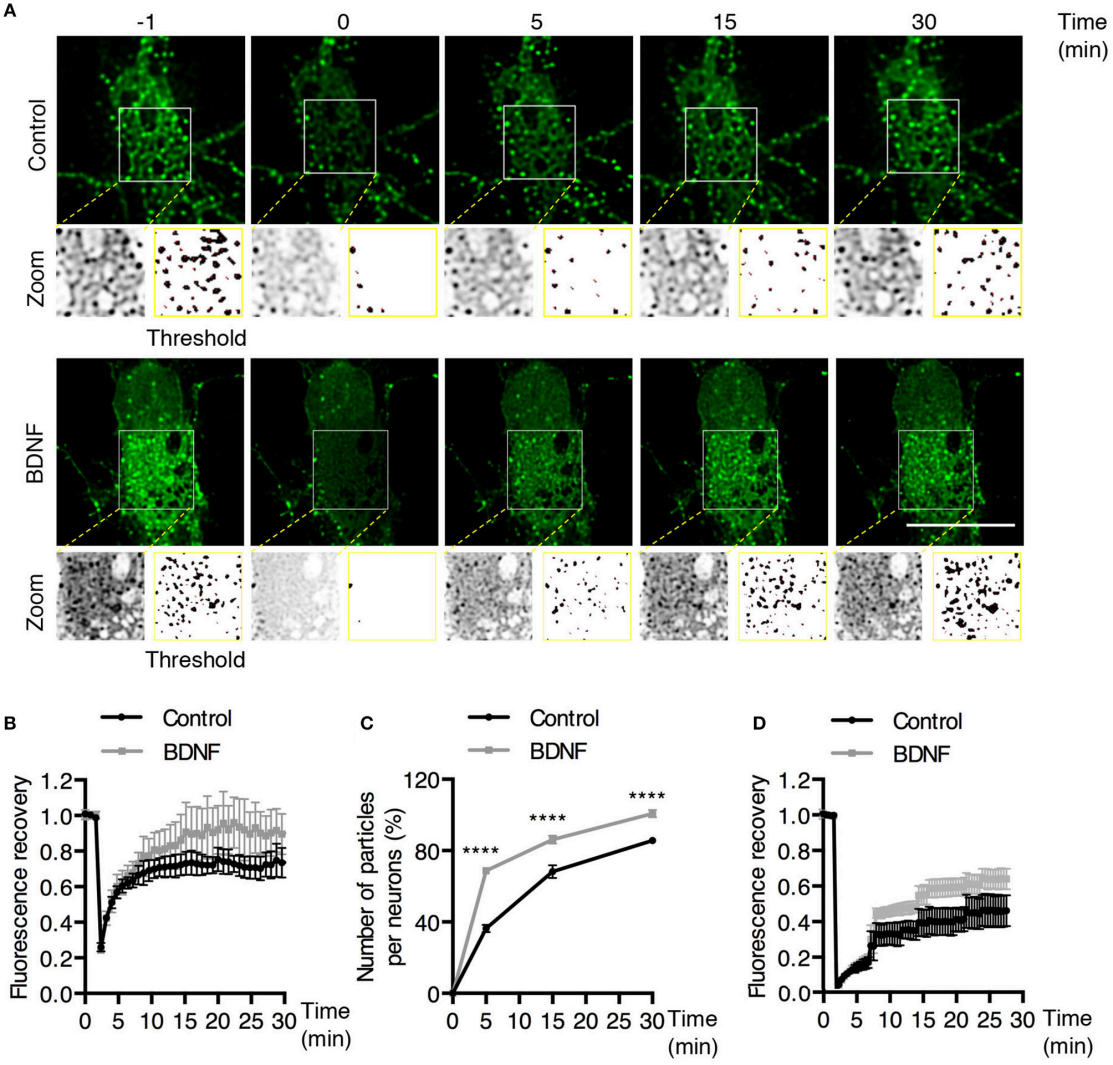
BDNF increases the recovery of Rab5-positive endosomes after photobleaching. **(A)** FRAP was performed in cell bodies of hippocampal neurons (time 0), and recovery was evaluated after 5, 15, and 30 min of photobleaching. Representative individual frame of a recording performed in a neuron expressing EGFP-Rab5. Recording was performed under control conditions or after BDNF treatment. The image shows endosome-like Rab5 particles in the cell body prior to FRAP (−1 min), during FRAP (0 min) and after FRAP (5, 15, 30 min). The white square indicated in the picture shows a zoomed-in image. On the right, a threshold image of the zoomed image (left) shows the endosomes quantified in **(C)**. **(B)** Time course of fluorescence recovery after FRAP of EGFP-Rab5-associated fluorescence in the photobleached soma. Black squares indicate the control neurons (n = 7) and gray squares the BDNF-stimulated neurons (*n* = 7). **(C)** The graph indicates the percentage (100% fluorescence was established at 1 min before FRAP) of endosome-like particles quantified (at *t* = 0, 5, 15, and 30 min) after FRAP. The black squares indicate the values obtained for the control neurons (*n* = 7), and gray squares indicate stimulated BDNF neurons (*n* = 7). **(D)** Time course of fluorescence recovery after FRAP of EGFP-Rab11-associated fluorescence in the photobleached soma. Black squares indicate the control neurons (*n* = 5) and gray squares the BDNF-stimulated neurons (*n* = 6). Four independent experiments were performed. Scale bar, 10 μm. The results are expressed as the mean ± SEM. ****p < 0.0001 by two-way ANOVA with Bonferroni's posttest.

To better understand the effect of BDNF on the mobility of EGFP-Rab5 endosomes in dendrites, we performed FRAP assays of hippocampal neurons stimulated with BDNF for 5 min. When a dendrite was photobleached, two populations of endosomes where observed: static (white and cyan arrowheads) and mobile (yellow arrowheads) (Figures 3A,B and Supplementary Video 1). Immediately after photobleaching, there was a recovery of cytoplasm-associated fluorescence, as shown with the blue arrow (Figure 3A). Then, an endosome derived from the static endosome, shown as the cyan arrowhead (blue arrow), recovered the fluorescence of the static photobleached endosome (yellow and white arrowheads in Figure 3A). Consistently, when the soma-associated fluorescence was bleached, it was possible to observe vesicles moving retrogradely toward the soma, as shown in the panels of Figure 4 and Supplementary Videos 2, 3. Altogether, these results suggest that by increasing the number and mobility of Rab5-positive endosomes, as shown in Figure 2, BDNF increases the retrograde transport of Rab5-positive endosomes to the soma. To study this possibility, we utilized FRAP assays of the complete cell bodies, including the initial segments of the dendrites, and studied the recovery of EGFP-Rab5 fluorescence in the soma of the cell bodies of cells treated with or without BDNF for 30 min. We noticed that there were two components in the EGFP-Rab5-associated fluorescence that were recovered after photobleaching. One accounted for the fluorescence associated with cytoplasmic EGFP-Rab5, and the other accounted for the fluorescence of EGFP-Rab5 associated with vesicles (Figure 3A, blue arrow and yellow arrowhead). When we quantified the FRAP in the cell body of EGFP-Rab5-transfected neurons, we did not observe changes in the kinetics of fluorescence recovery in neurons treated with BDNF compared to control neurons (Figure 5B). However, we observed that after BDNF stimulation, the recovery of Rab5-positive endosomes was faster than that in the control condition (Supplementary Videos 4, 5). Therefore, we applied a threshold to each image obtained after photobleaching, as indicated in Figure 5A, and quantified the fluorescence associated with EGFP-Rab5-positive vesicles. For these experiments, we considered the initial number of vesicles before the photobleaching as 100% of particles and then quantified the number of visible vesicles in the cell body after 5, 15, and 30 min of BDNF stimulation. We found that BDNF increased the number of vesicles in a time-dependent manner compared to the number observed in nontreated neurons (Figure 5A, yellow box in zoom, and Figure 5C). In addition, we repeated this protocol in neurons expressing EGFP-Rab11, and we observed that BDNF did not increase the recovery of EGFP-Rab11 fluorescence in the cell bodies (Figure 5D and Supplementary Figure 1). Because EGFP-Rab11 fluorescence in the cell bodies appeared to be less vesicular and less defined than EGFP-Rab5 fluorescence, we were unable to quantify discrete Rab11 endosomes (Supplementary Figure 1). Our experiments suggest that BDNF increases the transport of Rab5 endosomes toward the cell body.

**Figure 6:**
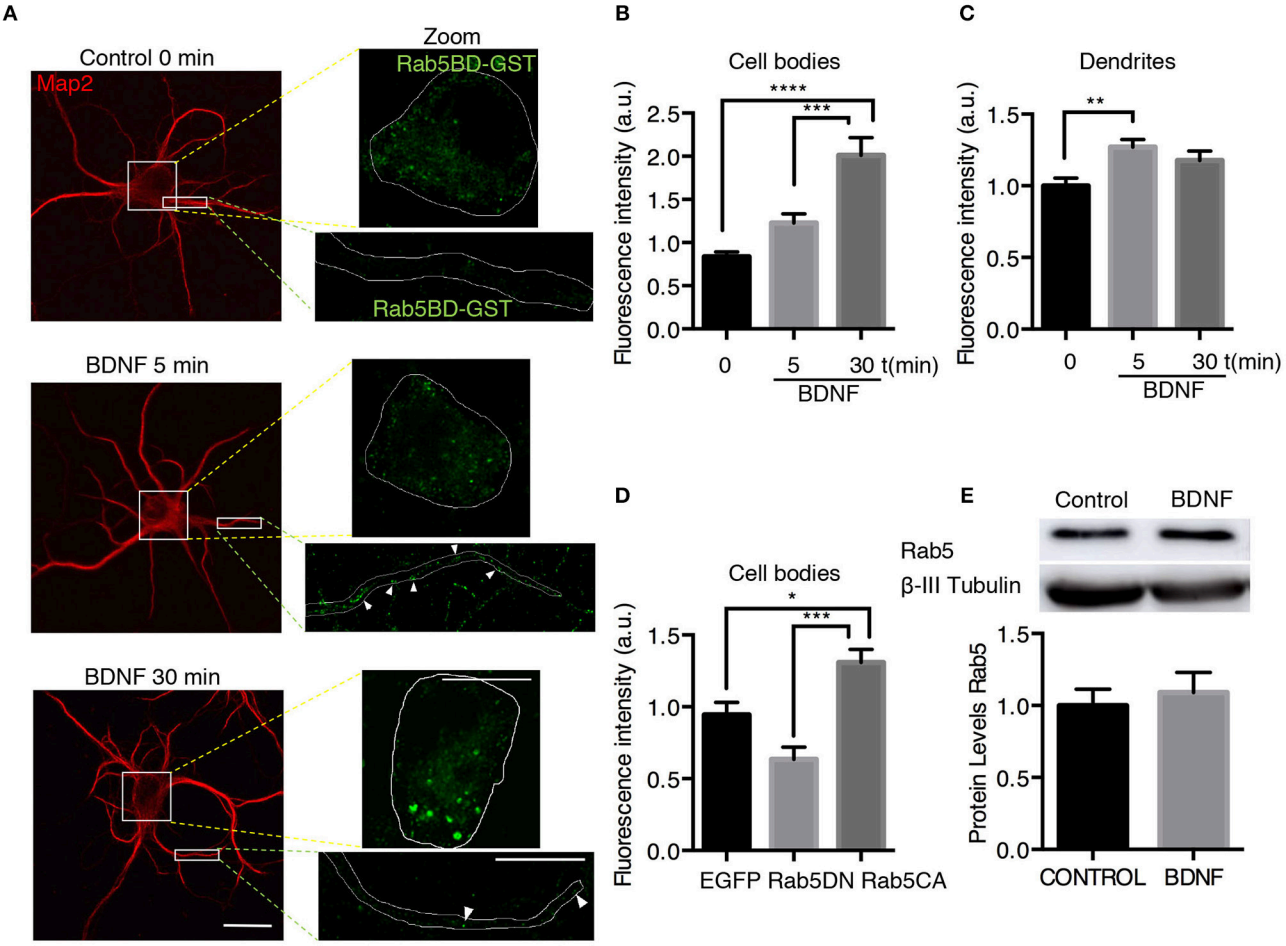
BDNF increases the activity of Rab5. **(A)** Neurons were nonstimulated (control time 0 min) or stimulated with BDNF for 5 min (BDNF 5 min) and 30 min (BDNF 30 min). Representative image of MAP2 immunostaining (red) and Rab5BD-GST (green). The white box indicates the region shown in the amplified photos of the cell body in the left panel and the dendrite shown in the right panel. Scale bar, 10 μm. **(B)** Quantification of fluorescence intensity in the cell body. **(C)** Quantification of fluorescence intensity in primary dendrites of control neurons and neurons treated for 5 and 30 min with BDNF. The results for the soma and dendrites were normalized to the values of control neurons (time 0 min). A total of 28–29 cell bodies and 66–87 dendrites were included from 4 independent experiments. **(D)** Quantification of fluorescence intensity in the cell bodies of 7 DIV neurons transfected with EGFP, EGFP-Rab5DN, or EGFP-Rab5CA. The results are expressed as the mean ± SEM. *p < 0.05; **p < 0.01; ***p < 0.001; ****p < 0.0001 by ANOVA with Bonferroni's posttest. **(E)** The Rab5a and ß-III tubulin levels of hippocampal neurons (9 DIV) stimulated with BDNF for 30 min. Lower part, quantification of the Rab5 levels of three independent experiments. The results are expressed as the mean ± SEM by Student's *t*-test.

To assess whether the increased TrkB/Rab5 colocalization and Rab5 mobility in dendrites and somas correlate with increased Rab5 activity after BDNF stimulation, we studied the distribution of active Rab5 *in situ* using the GST-fused with the Rab5 binding domain of Rabaptin5 (Rab5BD-GST), which specifically recognizes the GTP-bound active form of Rab5 (Wu et al., 2014). Using Rab5BD-GST as a probe of active-endogenous Rab5 (Rab5-GTP), followed by staining with an antibody against GST, we found that the treatment of neurons with BDNF for 5 and 30 min increased the amount of Rab5-GTP in the cell bodies and dendrites of hippocampal neurons in a time-dependent manner, with no changes in the levels of endogenous Rab5 measured by Western blotting (Figures 6A–C,E). In cell bodies, there was a significant increase in Rab5 activity after 30 min of BDNF stimulation that was not due to increased levels of Rab5 protein by BDNF treatment (Figures 6B,E). However, in dendrites, we observed increased levels of Rab5-GTP after only 5 min of BDNF stimulation (Figure 6D), similar to the results for TrkB and Rab5 colocalization in dendrites and cell bodies (Figure 1). As negative and positive controls for this experiment, we used hippocampal neurons transfected with a Rab5 dominant-negative (Rab5DN) or a Rab5 constitutively active (Rab5CA) mutant, respectively. Neurons expressing Rab5DN displayed significantly lower Rab5BD-GST labeling than neurons expressing Rab5CA (Figure 6D). Altogether, these results suggest that BDNF increases the activity of Rab5 in the soma and dendrites of hippocampal neurons in a spatial- and time-dependent manner.

### Long-Term Treatment of Hippocampal Neurons With BDNF Results in Increased Protein Levels of Rab5 and Rab11

**Figure 7:**
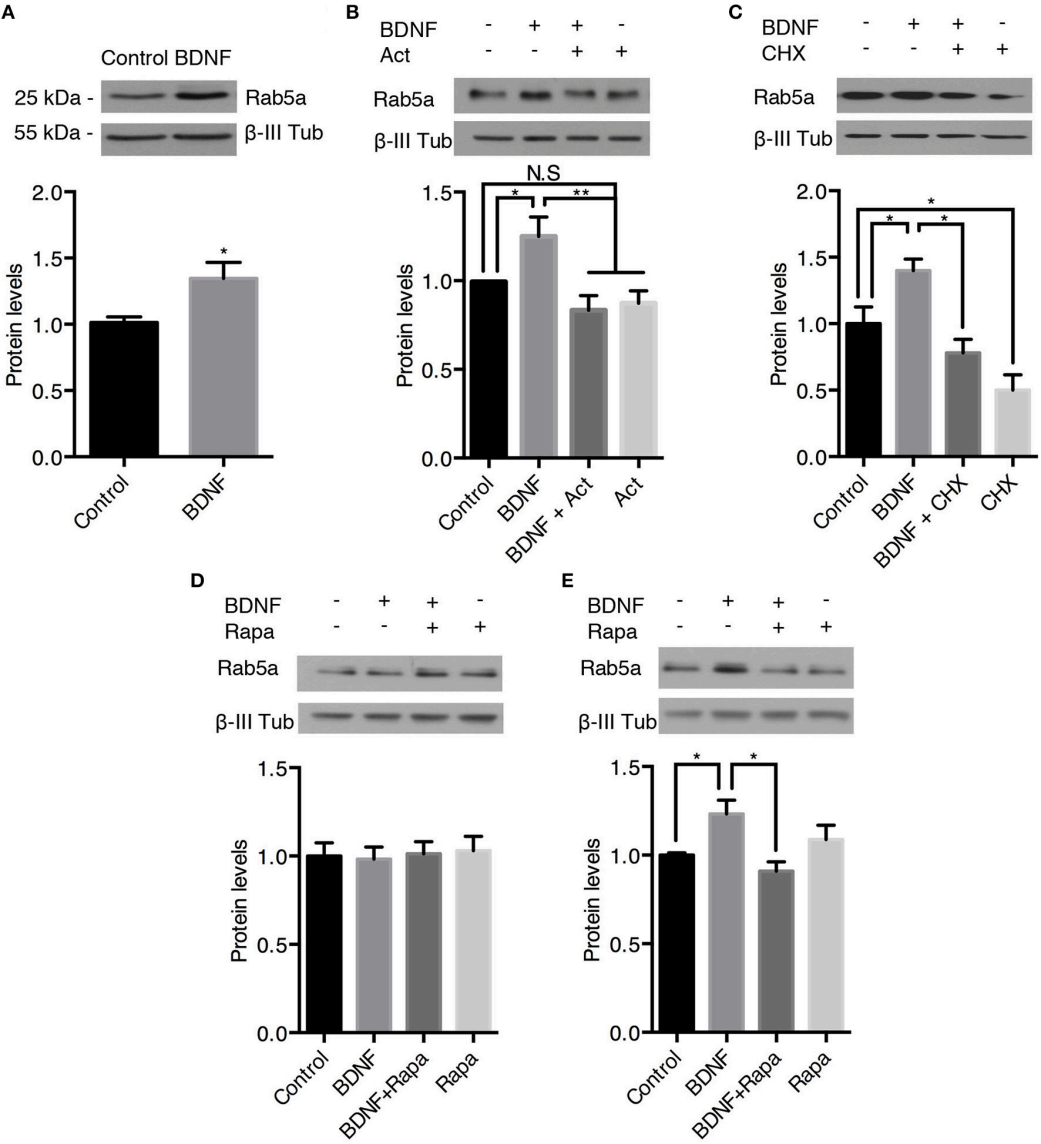
BDNF increases the protein levels of Rab5a. The Rab5a and ß-III tubulin levels in hippocampal neurons (9 DIV) stimulated with BDNF for 24 h **(A)** in the presence or absence of Actinomycin D **(B)** or cycloheximide (CHX) **(C)**. Bottom panel, densitometric quantification of the Ra5a levels normalized to the ß-III tubulin levels. The data represent 5 independent experiments. The results are expressed as the mean ± SEM. *p < 0.05; **p < 0.01 by ANOVA with Bonferroni's posttest. **(D,E)** Ra5a levels after 4 h **(D)** or 12 h **(E)** of treatment with rapamycin (Rapa) in the presence or absence of BDNF. Bottom panel, densitometric quantification of the Ra5a levels normalized to the ß-III tubulin levels. The data represent four independent experiments. The results are expressed as the mean ± SEM. *p < 0.05; **p < 0.01 by ANOVA with Bonferroni's posttest.

BDNF signaling increases protein levels by increasing transcription in a CREB-dependent manner downstream of PLC-gamma and ERK1/2 or translation in a mTOR-dependent manner downstream of PI3K and ERK1/2 signaling (Gonzalez et al., 2016). Therefore, we studied the effect of long-term administration of BDNF (4–24 h) on Rab5 and Rab11 protein levels. First, we studied whether the administration of BDNF for 4 or 12 h regulated the levels of the *rab5a* and *rab11a* genes. We found that BDNF increased the levels of *rab5a* in a time-dependent manner; an approximately 4-fold increase in *rab5a* was observed after 4 h of BDNF treatment (Supplementary Figure 2A), whereas after 12 h of BDNF stimulation, the levels of *rab5a* decreased to approximately 0.5-fold over the levels of the control (Supplementary Figure 2B). Conversely, the level of *rab11a* was unchanged by BDNF treatment at any of the time points studied (Supplementary Figures 2A,B). In this context, we first evaluated whether BDNF increases the protein levels of Rab5 after 24 h of BDNF treatment. We found that BDNF increased the level of Rab5a in approximately 20% of hippocampal neurons (Figure 7A) in a transcription- and translation-dependent manner, which is consistent with the results presented in Figures 7B,C, showing that actinomycin D and cycloheximide reduced the levels of Rab5 after BDNF treatment. Since BDNF increases protein translation in an mTOR-dependent manner (Takei et al., 2004), we evaluated whether the increase in Rab5a protein levels was sensitive to rapamycin, an mTOR pathway inhibitor (Schratt et al., 2004). We observed that 4 h of BDNF treatment did not affect the protein levels of Rab5; however, the presence of rapamycin decreased the increase in Rab5 protein levels caused by 12 h of BDNF treatment to the basal level (Figures 7D,E). These results indicate that BDNF regulates Rab5 protein levels by increasing or stabilizing its mRNA and by increasing its translation in an mTOR-dependent manner.

**Figure 8:**
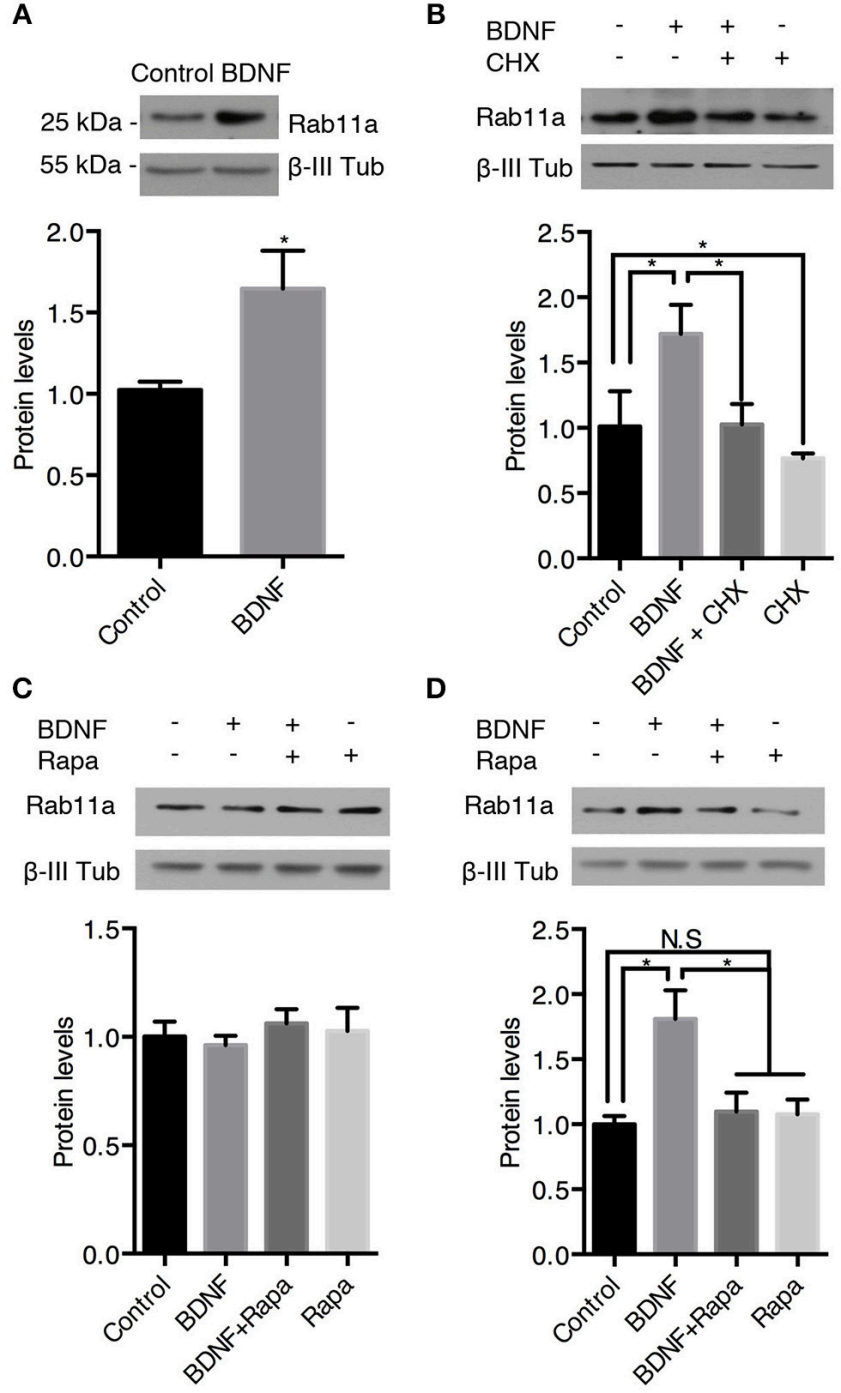
BDNF increases the protein levels of Rab11a. The Rab11a and ß-III tubulin levels in hippocampal neurons (9 DIV) stimulated with BDNF for 24 h **(A)** in the presence or absence of cycloheximide (CHX) **(B)**. Bottom panel, densitometric quantification of the Ra5a levels normalized to the ß-III tubulin levels. The data represent 5 independent experiments. The results are expressed as the mean ± SEM. *p < 0.05; **p < 0.01 by ANOVA with Bonferroni's posttest. **(C,D)** Ra11a levels after 4 h **(C)** or 12 h **(D)** of treatment with rapamycin (Rapa) in the presence or absence of BDNF. Bottom panel, densitometric quantification of the Ra11a levels normalized to the ß-III tubulin levels. The data represent 4 independent experiments. The results are expressed as the mean ± SEM. *p < 0.05 by ANOVA with Bonferroni's posttest.

We also analyzed the protein levels of Rab11 upon BDNF treatment. Similar, to the findings for Rab5, BDNF increased the level of Rab11 after 24 h of treatment; this effect was abolished by cycloheximide (Figures 8A,B). In contrast, 4 h of BDNF treatment did not affect the protein level of Rab11 (Figure 8C), while 12 h of BDNF treatment increased the level of Rab11 to values similar to 24 h of treatment (Figure 8D). In addition, the BDNF effect on the Rab11 protein levels (12 h treatment) was diminished by rapamycin (Figure 8D). All together, these results indicate that BDNF regulated the levels of both the Rab5 and Rab11 GTPases at the translational level in an mTor-dependent manner.

### Long-Term Treatment of Hippocampal Neurons With BDNF Results in Increased Dendritic Branching That Is Impaired by Reducing the Activity of the Rab5 and Rab11 Proteins

**Figure 9:**
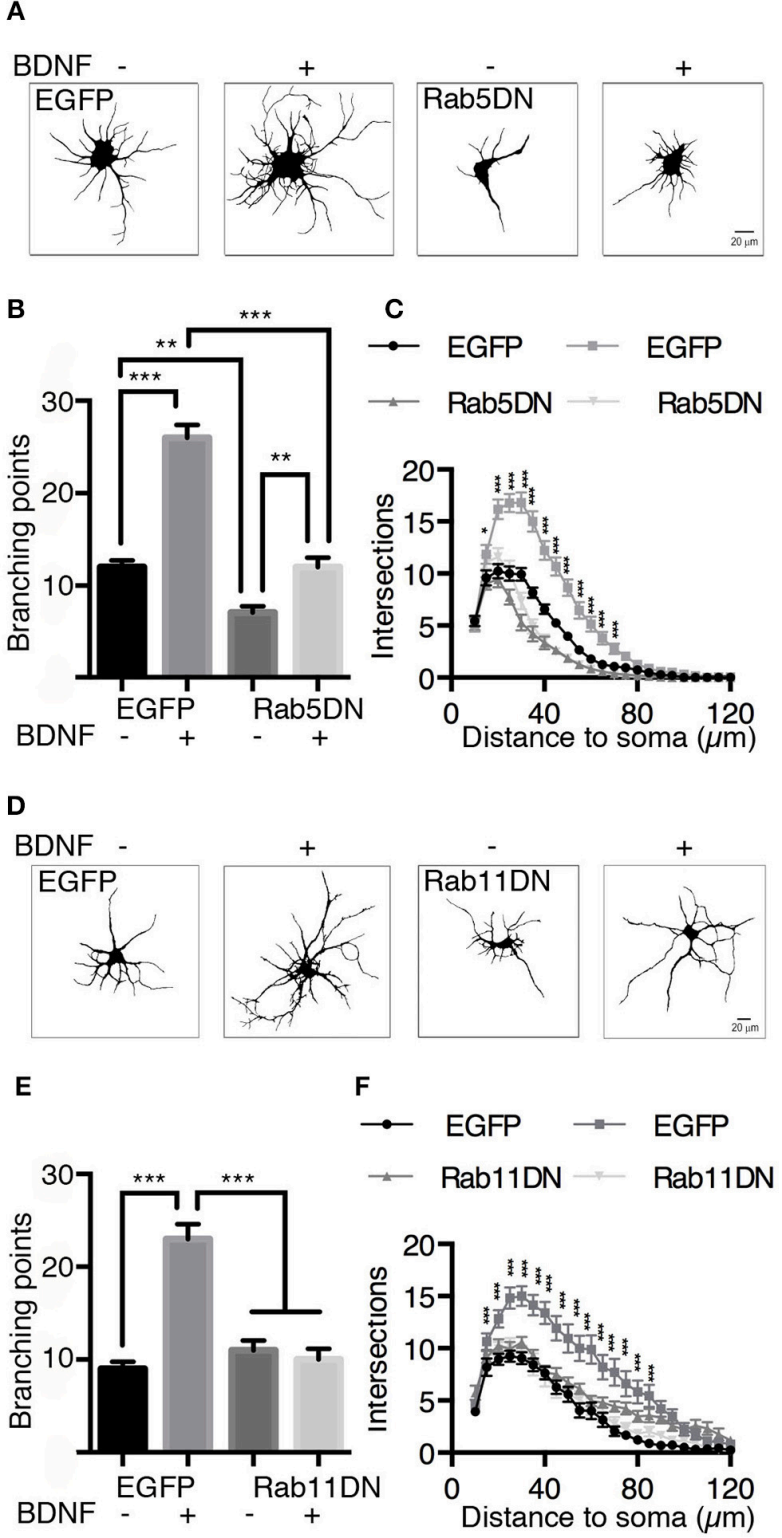
The expression of dominant-negative mutants of Rab5 (Rab5DN) decreases BDNF-induced dendritic branching in hippocampal neurons. **(A)** Representative image of hippocampal neurons (7 DIV) transduced with adenovirus EGFP (in A at the left) or with a dominant-negative mutant of Rab5 fused to EGFP (Rab5DN in **A** at the right) and stimulated with BDNF for 48 h. After fixation, the neurons were labeled with anti-MAP2, observed by fluorescence microscopy and subjected to morphometric analysis. **(B)** Sholl analysis of the arborization profiles of neurons expressing EGFP and Rab5DN-EGFP in the presence or absence of BDNF. **(C)** Quantification of the branching points of neurons that overexpressed Rab5DN or EGFP and exposed to BDNF. *N* = 27–34 neurons from 3 different experiments. **(D)** Representative image of hippocampal neurons (7 DIV) that were transduced with adenovirus EGFP (in **D** at the left) or a dominant-negative mutant of Rab11 fused to EGFP (Rab11DN in **A** at the right) and stimulated with BDNF for 48 h. After fixation, the neurons were labeled with anti-MAP2, observed by fluorescence microscopy and subjected to morphometric analysis. **(E)** Sholl analysis of the arborization profiles of neurons expressing EGFP and Rab11DN-EGFP in the presence or absence of BDNF. **(F)** Quantification of the branching points of neurons that overexpressed Rab11DN or EGFP exposed to BDNF. *N* = 16–19 neurons from 3 different experiments. The results are expressed as the mean ± SEM. *p < 0.05; **p < 0.01; ***p < 0.001 by two-way ANOVA with Bonferroni's posttest.

It is well known that BDNF induces an increase in dendritic branching both *in vivo* and *in vitro* (Gonzalez et al., 2016). To evaluate whether Rab5 activity is required for BDNF-induced dendritic branching in hippocampal neurons, we stimulated neurons expressing EGFP or the dominant-negative mutant of Rab5 (EGFP-Rab5DN) with BDNF for 48 h. We found that the expression of EGFP-Rab5DN produced a change in the morphology of the somato-dendritic arbor in comparison with neurons that only expressed EGFP (Figure 9A). Using Sholl analysis and the quantification of branching points, we found that the expression of Rab5DN reduces the branching points compared to the control condition (Figures 9A–C). Although neurons expressing Rab5DN responded to BDNF by increasing the number of primary dendrites, they were not able to respond to the same extent as neurons expressing EGFP and treated with BDNF, which showed an increase in branching points in addition to an increase in the number of primary dendrites (Figure 9C). These results are somehow different from those observed when neurons express a dominant-negative mutant for Rab11 (Rab11DN). Similar to our previous observations (Lazo et al., 2013), neurons expressing Rab11DN have a similar number of dendrites to neurons expressing EGFP. However, they did not respond to BDNF (Figures 9D–F). These results indicate that Rab5 activity is required for the maintenance of dendritic arbors *in vitro*, and although EGFP-Rab5-expressing neurons responded to BDNF by increasing the number of primary dendrites, Rab5 activity is required to observe the effect of BDNF on the branching of higher level dendrites.

## Discussion

Several lines of evidence have consistently shown that the internalization and transit of Trk receptors through the endocytic pathway are required for proper signaling and neuronal function (Bronfman et al., 2014; Cosker and Segal, 2014). The mechanism by which neurotrophin receptors use the endosomal pathway for signaling in neurons is well documented. For example, “signaling endosomes” containing ligand-bound neurotrophin receptors have been extensively described for axon-to-nucleus communication in peripheral neurons (Bronfman et al., 2003; Delcroix et al., 2003; Harrington et al., 2011). Additionally, BDNF signaling endosomes have been described to have a role in dendrite-to-nucleus communication in central neurons (Cohen et al., 2011). However, how neurotrophins regulate the endosomal system for proper signaling is just beginning to be understood (Cosker and Segal, 2014). Rabs are monomeric GTPases that act as molecular switches to regulate membrane trafficking. They achieve this function by binding a wide range of effectors that include SNAREs, signaling molecules and molecular motors. Among the Rab GTPases, Rab5 is the key GTPase regulating early endosomes and the first endocytic station of endocytosed receptors (Stenmark, 2009). Of note, several lines of evidence have shown that there is crosstalk between Rab5 activity and tyrosine kinase signaling receptors (Chiariello et al., 1999; Jozic et al., 2012; Ong et al., 2014). Our aim was to study the regulation of the Rab5-positive endosomes in relation to BDNF and at different levels, including dynamics, activity and protein levels in hippocampal neurons. We found that BDNF increased the colocalization of TrkB in dendrites and cell bodies, increasing the vesiculation of Rab5-positive endosomes in the somatodendritic compartment. These findings correlated with the increased mobility of Rab5 endosomes in dendrites and increased the movement of Rab5 endosomes from dendrites to the cell body. Consistently, BDNF induced an early activation of Rab5 in dendrites (5 min) followed by increased activation of Rab5 in cell bodies (30 min). Long-term treatment of hippocampal neurons with BDNF (12–24 h) increased the protein levels of Rab5 and Rab11 in an mTOR-dependent manner. Finally, expression of a dominant-negative mutant of Rab5 reduced the basal arborization of nontreated neurons and BDNF-induced arborization. We propose that BDNF increases the activity of Rab5 in dendrites to foster local dendritic growth and to increase BDNF signaling propagation to the cell soma.

We have previously shown that BDNF/TrkB increases the activity of Rab11 in dendrites of hippocampal neurons by increasing local recycling and thus signaling of BDNF (Lazo et al., 2013). Rab5 regulates the fusion of endocytosed vesicles to form early endosomes where receptors are sorted to the recycling pathway that is regulated by Rab11. Here, we show that BDNF signaling also regulates the activity of Rab5, suggesting that BDNF in dendrites increases the activity of both GTPases to increase the early recycling pathways for local signaling. One intriguing aspect of our research, however, is that the mobility of both endosomes was oppositely regulated by BDNF. While BDNF decreases the mobility of Rab11 to allow local recycling (Lazo et al., 2013), it increases both the number and mobility of Rab5-positive vesicles in dendrites (current study). The movement of Rab5 vesicles increased in both the anterograde and retrograde directions. However, Rab5 movements were biased to the retrograde direction, consistent with a study indicating that 60% of microtubules are oriented with the minus end toward the soma in mammalian cells (Ayloo et al., 2017). It is possible that while anterograde movement of Rab5-positive vesicles is required for dendritic growth, retrograde movement of Rab5 resulted in increased levels of Rab5-positive vesicles in the cell body. Consistently, we showed by live-cell microscopy that Rab5-positive endosomes moved from primary dendrites to the cell body. While performing live-cell microscopy of dendritic EGFP-Rab5 transfected neurons after photobleaching, we observed that Rab5-associated fluorescence recovered in the same place, in addition to the observed mobile vesicles (Figure 3), suggesting that we monitored both stationary and mobile early endosomes. Altogether, our research suggests that BDNF defines a different population of Rab5 early endosomes that sort components to the recycling pathway for local recycling, and another population engages in long-distance trafficking to the soma or to distal dendrites. It is possible that a coordinated action of actin-based motors regulates local trafficking of signaling receptors since both Rab5 and Rab11 interact with myosin proteins to coordinate local membrane trafficking (Schafer et al., 2014; Sui et al., 2015; Masters et al., 2017).

Different lines of evidence have shown that both dynein and neuronal kinesin KIF21B engage TrkB-BDNF for long-distance trafficking in dendrites (Ghiretti et al., 2016; Ayloo et al., 2017). On the other hand, active Rab5 has been described to bind the Hook-interacting protein complex, which interacts with dynein and dynactin to regulate the retrograde transport of axonal proteins in neurons (Guo et al., 2016). Additionally, there is evidence that dynein and dynactin contribute to 85–98% of long-inward translocation of Rab5 early endosomes in HeLa cells (Flores-Rodriguez et al., 2011). Of note, dynein-mediated transport of Rab5-positive early endosomes is required for dendritic branching in *Drosophila melanogaster* dopamine neurons (Satoh et al., 2008). These results are consistent with our findings showing that Rab5 activity is required for the stability of dendrites and BDNF-mediated dendritic branching in hippocampal neurons (Figure 7). Altogether, these results suggest that microtubule-associated molecular motors, most likely dynein, drive the long-distance movement of Rab5 endosomes from dendrites to the soma in response to BDNF, which is a process required for dendritic branching. This process might be important in dendrite-to-nucleus communication as suggested by the results showing that dendritic BDNF increases expression of the immediate early genes c-fos and Arc in the cell bodies of both hippocampal and striatal neurons (Cohen et al., 2011; Liot et al., 2013).

**Figure 10:**
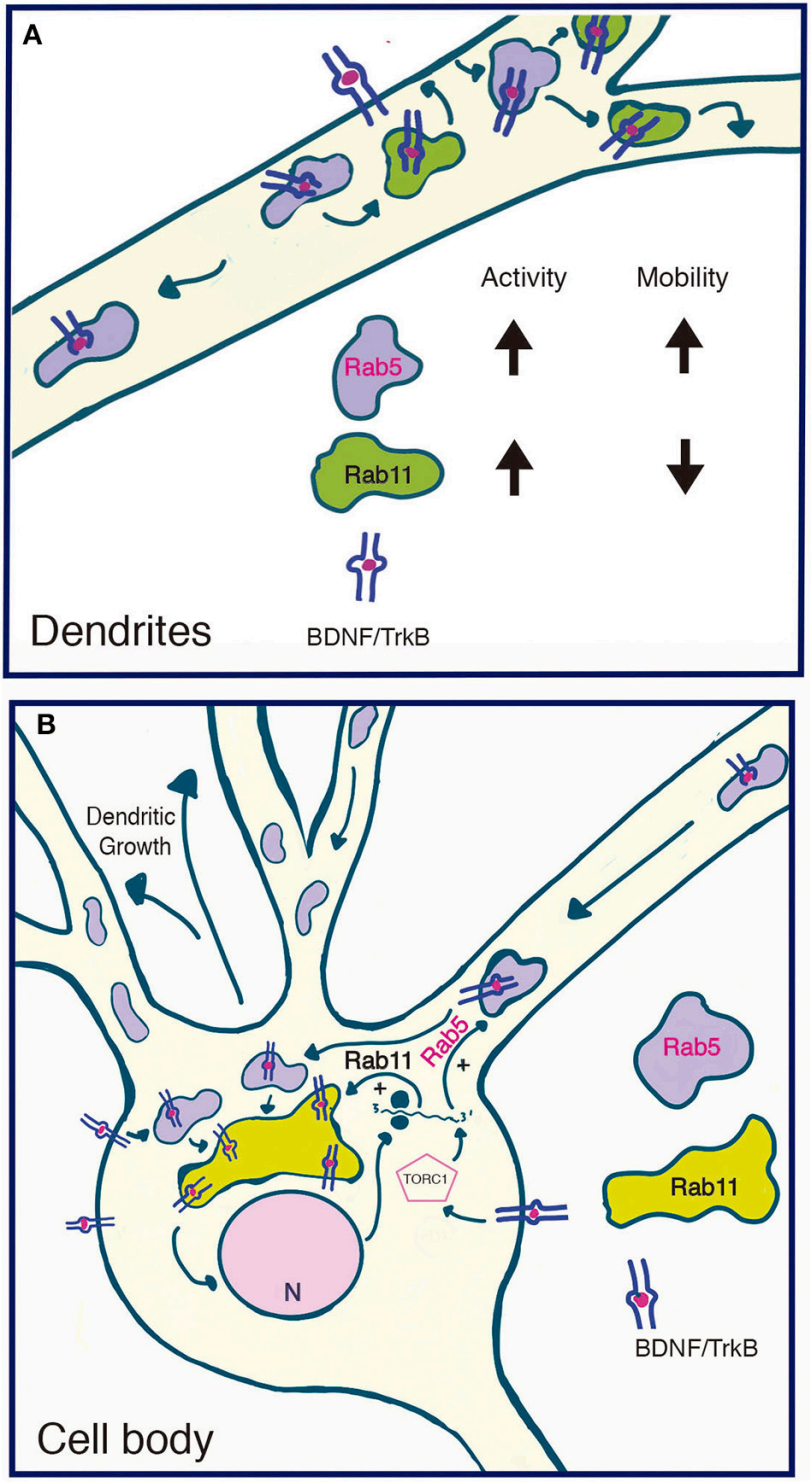
Model summarizing the functional relationship between the early recycling pathway and BDNF/TrkB signaling. **(A)** In dendrites, BDNF increases the activity and reduces the mobility of Rab11-endosomes, fostering the local recycling of TrkB in dendrites and increasing local BDNF signaling (Lazo et al., 2013). On the other hand, BDNF transiently increases the activity of Rab5, a process that increases the number and mobility of Rab5 endosomes in dendrites. Since Rab5 early endosomes are upstream of Rab11 recycling endosomes, increased activity of Rab5 may, on the one hand, foster the Rab11-dependent recycling of TrkB in dendrites and, on the other hand, increase the transport of Rab5 endosomes to the soma. **(B)** In the soma, increased activity of both Rab5 (this paper) and Rab11 (Lazo et al., 2013) by BDNF may increase the cell body recycling of TrkB, increasing the long-lasting signaling of BDNF required for dendritic growth. Increased activity of the TORC1 complex by BDNF increased the protein levels of Rab5 and Rab11 acting as a positive feedback loop that contributes to BDNF-induced dendritic branching.

We also observed that BDNF increases Rab5 vesiculation and the number of Rab5-positive endosomes in dendrites, a process that correlates with increased vesicles containing active Rab5. Fusion and fission events are required for proper early endosome function and sorting of endocytosed receptors and ligands (Skjeldal et al., 2012). Rab5 regulates these process by regulating fusion of newly endocytosed receptors to form the early or sorting endosomes; from there, fission events allow sorting into the endocytic pathways (Driskell et al., 2007). It is possible that BDNF increases fusion and fission events to increase the vesiculation of Rab5 in dendrites, or increases the recruitment of cytosolic inactive Rab5 to newly formed or preexisting endosomes (Figure 10). However, direct evidence of these phenomena remain to be analyzed by a more refined technique such as fluorescence resonance energy transfer as performed by Verboogen to visualize SNARE trafficking and fusion (Verboogen et al., 2017).

Like other Rab proteins, Rab5 activity and localization is regulated by GEFs, GAPs and different effectors (Zerial and McBride, 2001; Stenmark, 2009). Different extracellular cues have been described to regulate Rab5 activity. For instance, in PC12 cells, NGF activation of TrkA recruits RabGAP5, which inactivates Rab5, producing a delay in the maturation of signaling endosomes and prolonging signaling and neurite outgrowth in PC12 cells (Liu et al., 2007). Consistently, expression of a dominant-negative Rab7 in PC12 cells enhances NGF-mediated signaling (Saxena et al., 2005) while it abolishes axonal retrograde transport of TrkB-positive endosomes in motor neurons (Deinhardt et al., 2006). On the other hand, in cortical neurons, semaphorin 3A increases the activity of Rab5 in axons to promote growth cone collapse (Wu et al., 2014), suggesting that the activation of Rab5 might induce different outcomes depending on the extracellular cues, the signaling pathways activated and the neuronal processes that are regulated. We observed that BDNF increases Rab5 activity and that these processes are required for BDNF-dependent dendritic arborization, suggesting that in hippocampal neurons, BDNF-mediated activation of Rab5 is required for proper signaling, contrary to the results observed in PC12 cells (Liu et al., 2007). One question that arises is how BDNF regulates the activity of Rab5. There are no antecedents that could lead us to hypothesize a direct effect of TrkB signaling on Rab5 activity. However, we could speculate that by phosphorylating Rab5, BDNF modulates its interaction with GEFs, thus increasing its activation. In support of this speculation is the fact that different kinases including ERK1, a BDNF/TrkB downstream kinase, phosphorylate Rab5 (Chiariello et al., 1999), and Rin1, a GEF for Rab5, has been associated with other RTKs to increase Rab5 activity (Hunker et al., 2006). The activation of Rab5 should be a tightly regulated process, and we observed that the activation of Rab5 induced by BDNF is time dependent without promoting sustained activation. Sustained activation of Rab5 in axons disrupts retrograde axonal trafficking of NGF signals in basal forebrain cholinergic neurons, suggesting that Rab5 activity must be tightly regulated for proper neuronal function (Xu et al., 2016). Overactivation of Rab5 could be deleterious for neurons. Indeed, we have observed that expression of Rab5CA induces neurodegeneration in hippocampal cultures (data not shown), a phenomenon that it is not observed when hippocampal neurons are transduced with Rab11CA (Lazo et al., 2013).

Our results indicate that both Rab5 and Rab11 activity are required for BDNF-induced dendritic branching, indicating that the transit and correct endosomal sorting of BDNF receptors are required for proper signaling. For example, retrolinkin, a receptor that tethers vesicles, interacts with endophilin A1, a protein involved in generating endocytic necks, which is recruited to the early endosomal compartment in response to BDNF (Burk et al., 2017). Both proteins are required for BDNF early endocytic trafficking and spatiotemporal regulation of BDNF-induced ERK activation (Fu et al., 2011).

Finally, we found that BDNF increases both the mRNA and proteins levels of Rab5a in an mTOR-dependent manner. The activation of mTOR kinase has been described as a key signaling pathway regulating the translation of proteins mediated by BDNF (Leal et al., 2014). Specificity is achieved because BDNF, in addition to regulating translation, induces a specific miRNA-dependent repression (specific miRNA downregulation) and stabilizes the Dicer-TRBP complex, increasing global maturation of miRNA (Ruiz et al., 2014). The fact that the protein levels of Rab5 and Rab11 are upregulated by BDNF in an mTOR-dependent manner suggests that the specific growth program initiated by BDNF acts as a positive feedback loop to increase BDNF- and Rab5-Rab11-dependent dendritic growth. Indeed, mTOR activation is required for dendritic arborization of central neurons (Jaworski et al., 2005) and, consistently, both Rab5 and Rab11 activity is required for BDNF-induced neuronal growth.

Our results and those of others allow us to propose a model (Figure 10) addressing the functional role between the early recycling pathway regulated by Rab5-Rab11 and BDNF/TrkB signaling in neurons. We propose that BDNF is able to regulate the endosomal system by regulating the activity of Rab5 and Rab11 in a time- and space-dependent manner. This process allows both increased local signaling in dendrites and increased signaling in cell bodies. While this model predicts two different populations of recycling endosomes (dendritic Rab11 endosomes vs. the perinuclear cell body Rab11 recycling endosome), the early endosomal pathway might be coordinating dendritic and cell body signaling.

Altogether, our results suggest that Rabs are key proteins that regulate BDNF signaling, and further research is required to better understand the mechanism that leads to BDNF-mediated activation of Rab5 and Rab11 and how this process is coordinated with molecular motors for both local and long-distance signaling of BDNF.

## Author Contributions

GM-A performed and designed experiments and drafted the manuscript. AG performed and designed experiments and drafted part of section Materials and Methods and Figure Legends. NS performed experiments and FB supervised the experimental design and drafted the article.

### Conflict of Interest Statement

The authors declare that the research was conducted in the absence of any commercial or financial relationships that could be construed as a potential conflict of interest.
