# Supplementary material for: Brain-Derived Neurotrophic Factor (BDNF) Regulates Rab5-Positive Early Endosomes in Hippocampal Neurons to Induce Dendritic Branching
Source: Front Cell Neurosci. 2018 Dec 17;12:493. doi: 10.3389/fncel.2018.00493 (PMC6304382; doi:10.3389/fncel.2018.00493)
Supplement: Supplementary file 6 [file Data_Sheet_1.pdf]

Supplementary Figures

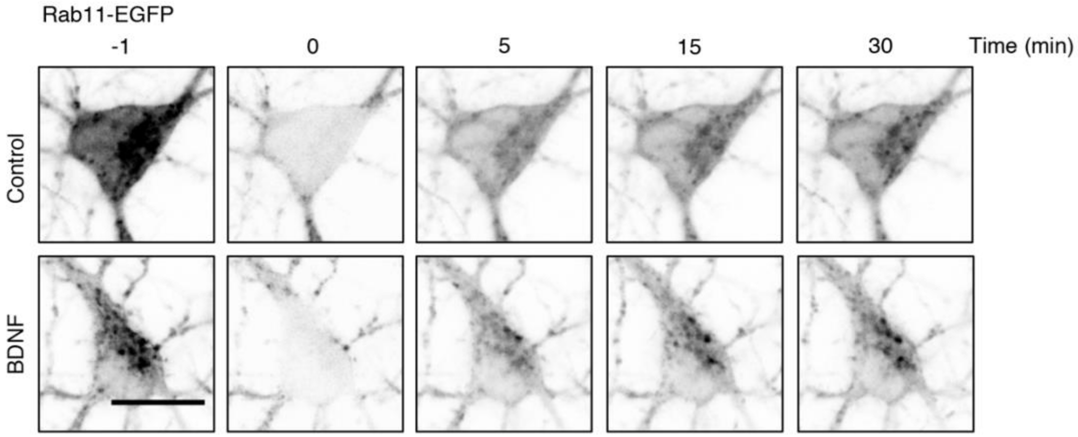

Supplementary Figure 1. FRAP in the cell body of EGFP-Rab11. FRAP was performed in cell bodies of hippocampal neurons (time 0), and recovery was evaluated after 5, 15 and 30 min. Representative individual frames of a recording performed in a neuron expressing EGFP-Rab11. Recording was performed in control conditions or after BDNF treatment. The panel shows EGFP-Rab11 endosomes in the cell body before FRAP (-1 min), just after FRAP (0 min) and after 5, 15, 30 min of FRAP.

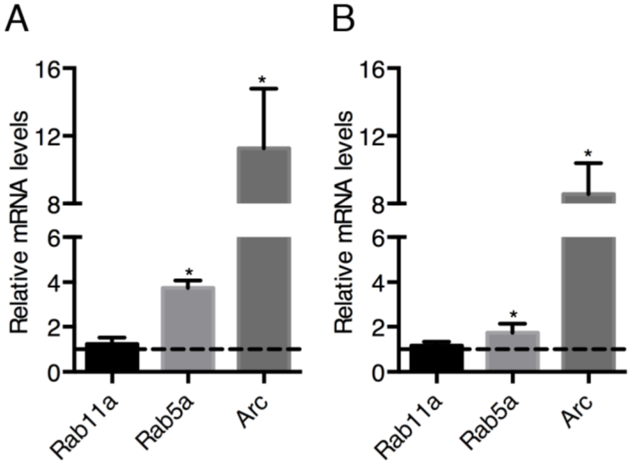

Supplementary Figure 2. BDNF stimulation increases rab5a but not rab11a mRNA levels. A total of 8-9 DIV hippocampal neurons were stimulated with BDNF (50 ng/mL) for 4 h (A) or 12 h (B). mRNA levels of rab5a, rab11a and arc were analyzed by qPCR. Expression levels were normalized by β-actin, Tbp and Pgk-1 mRNA levels. Arc mRNA levels were evaluated as a positive control of BDNF stimulation. Quantification was done of relative levels of mRNA with $2^{-\Delta\Delta Ct}$, showed as a fold changes of nonstimulated neurons (black dashed line). The results are expressed as the mean ± SEM, from 3 independent experiments. *<0.5; by Student's T-test.
