# Supplementary material for: Brain-Derived Neurotrophic Factor (BDNF) Regulates Rab5-Positive Early Endosomes in Hippocampal Neurons to Induce Dendritic Branching
Source: Front Cell Neurosci. 2018 Dec 17;12:493. doi: 10.3389/fncel.2018.00493 (PMC6304382; doi:10.3389/fncel.2018.00493)
Supplement: Supplementary file 7 [file Data_Sheet_2.pdf]

Supplementary Video description

Video 1. FRAP of EGFP-Rab5 in primary dendrites. Hippocampal neurons transfected with EGFP-Rab5 (in green) were stimulated with BDNF, and a FRAP assay was performed in a primary dendrite. The video shows endosome-like Rab5 particles (green) in dendrites prior to FRAP, during FRAP and after FRAP (until 300 s). White arrowhead shows a static endosome photobleached, which is recovered. Cyan arrowhead shows a static endosome that generate the yellow arrowhead endosome. Yellow arrowhead shows mobile endosome that arrived to the photobleached endosome.

Video 2. Retrograde transport of Rab5-EGFP to the cell body. Hippocampal neurons transfected with EGFP-Rab5 (in black) were stimulated with BDNF, and a FRAP assay was performed in the cell body. Fluorescence of EGFP-Rab5 was oversaturated to allow the observation of endosomes movement.

Video 3. Retrograde transport of Rab5-EGFP to the cell body. Retrograde labels of endosomes in video 2; each color is a different retrograde-transported endosome.

Video 4. FRAP assay of EGFP-Rab5 in non-stimulated neurons. Time lapse of hippocampal neuron transfected with EGFP-Rab5 (in black) photobleached in the cell body in control condition (non-stimulated). Recovery of Rab5 positive endosomes are observed after photobleaching the soma. Fluorescence of EGFP-Rab5 was oversaturated to allow the observation of endosomes.

Video 5. FRAP assay of EGFP-Rab5 in BDNF stimulated neurons. Time lapse of hippocampal neuron transfected with EGFP-Rab5 (in black) photobleached in the cell body after 5 minutes of BDNF stimulation. Recovery of Rab5 positive endosomes are observed after photobleaching the soma. Fluorescence of EGFP-Rab5 was oversaturated to allow the observation of endosomes.
